# Fullerene-Containing Electrically Conducting Electron Beam Resist for Ultrahigh Integration of Nanometer Lateral-Scale Organic Electronic Devices

**DOI:** 10.1038/s41598-017-04451-9

**Published:** 2017-06-27

**Authors:** Anri Nakajima, Tetsuo Tabei, Tatsuya Yasukawa

**Affiliations:** Research Institute for Nanodevice and Bio Systems, Hiroshima University, 1-4-2 Kagamiyama, Higashihiroshima, Hiroshima, 739-8527 Japan

## Abstract

An outstanding issue with organic devices is the difficulty of simultaneously controlling the lateral size and position of structures at submicron or nanometer scales. In this study, nanocomposite electron beam (EB) organic resists are proved to be excellent candidates for electrically conductive and/or memory component materials for submicron or nanometer lateral-scale organic electronic devices. The memory and the resist patterning characteristics are investigated for a positive electron beam resist of ZEP520a containing [6,6]-phenyl-C_61_ butyric acid methyl ester (PCBM). Regarding the memory characteristics, good programming and excellent retention characteristics are obtained for electrons. The carrier transfer and retention mechanisms are also investigated. Regarding the resist patterning characteristics, it is found that line patterns (square patterns) of ZEP520a containing PCBM can be made with widths (side lengths) of less than 200 nm by using an extremely simple process with only EB exposures and developments. The distribution of PCBM molecules or their aggregations is also clarified in ZEP520a containing PCBM. The results of this study open the door to the simple fabrication of highly integrated flexible memories and electrical wires as well as of single-electron or quantum devices, including quantum information devices and sensitive biosensors for multiplexed and simultaneous diagnoses.

## Introduction

In the field of large scale integrated circuits (LSIs), various techniques of fabricating nanoscale structures have been established for dense integration of devices with low power consumption. For example, flash-memory-type three-terminal devices are typical nonvolatile memories in LSIs, and nanoscale floating dot structures have been developed for higher levels of integration^1–4^. In addition, nanoscale dot and wire structures have been developed that enable quantum and single electron devices to be incorporated in LSIs^5–7^. Even high sensitive biosensors based on Si nanowire-channel transistors and single-electron transistors have been developed for integrated single-chip sensor systems suitable for multiplexed and simultaneous diagnoses^8–11^. Moreover, semiconductor double quantum dots are expected to be an essential building block to form a solid-state based quantum bit (qubit) for quantum information^12^. Among the various fabrication techniques, top-down fabrication using electron beam (EB) lithography makes it possible to make various nanoscale structures and high-performance devices.

On the other hand, organic electrical and optical devices have attracted much attention because of their unique advantages of low weight, high mechanical flexibility, cost effectiveness, and good chemical structural versatility compared with inorganic devices^13–19^. However, most of these devices are over a micrometer in lateral size. An outstanding issue with organic devices is the difficulty of simultaneously controlling the lateral size and position of structures in the submicron or nanometer region. Conventional EB lithography techniques have many problems when they are used for the above purposes. For example, it is difficult to remove only the mask organic resist material by using oxygen plasma ashing or cleaning with a sulfuric acid hydrogen peroxide mixture because of the small selective etching ratio between the resist and the organic etching object. It is also difficult to obtain a large selective ratio in dry etching between the two.

In regions with lateral dimensions larger than 5 μm, in contrast, photosensitive electrically conductive organic polymer composites have been reported. They consist of a photopolymerizable matrix allowing the material to be lithographically patterned with photons. In particular, high-aspect ratio structures with a minimum lateral resolution of 5 μm have been fabricated using an electrically conductive photosensitive negative resist of SU8 including silver particles and ultraviolet light^20, 21^. In addition, a pattern with a line width of 10 μm was obtained with an electrically conductive SU8 resist embedding carbon black particles^22^. These resist materials are electrically conductive themselves, and consequently, it is not necessary to use an additional resist for determining their lateral sizes and positions.

In addition, nanocomposite organic resist polymers in which fullerenes are incorporated in a conventional EB resist of ZEP520 have been reported^23–25^. However, they have only been used as a mask resist with improved dry etching resistance for submicron lateral-scale patterning. They have not been used as carrier conducting materials for electronic devices, and their characteristics related to carrier transport have not been investigated yet. On the other hand, there have been reports indicating that gate insulating organic polymers containing fullerene molecules, referred to as nanocomposite organic polymers hereafter, are promising materials for organic flash memories because of their relatively simple fabrication technique^26–31^. Since it is suggested that electrons or holes are injected into and stored in the LUMO or HOMO levels of the fullerenes and the carriers are transferred through the levels in the nanocomposite organic polymers, there is a possibility of creating EB-patternable electrically conductive nanocomposite organic polymers having submicron or nanometer lateral sizes if organic EB resists can be used for the matrix polymers.

Therefore, the purpose of this study was to determine whether such nanocomposite organic EB resist polymers are promising electrically conductive and/or memory component materials for submicron and nanometer lateral-scale electronic devices. Here, we used a positive-type EB resist of ZEP520a as the matrix organic polymer and incorporated fullerenes. ZEP520a is a copolymer of methyl α-chloroacrylate and α-methylstyrene. We chose [6,6]-phenyl-C_61_ butyric acid methyl ester (PCBM) to be the fullerene incorporated in the resist. We examined the memory and resist patterning characteristics of the nanocomposite organic-resist polymer. It should be noted that ours is the first report on the electrical conducting and memory characteristics or the resist patterning characteristics of ZEP520a containing PCBM. Regarding the memory characteristics, flatband voltage shifts Δ*V*_*F*_ having substantial magnitudes (|Δ*V*_*F*_|) were observed after applying a negative programming voltage to the capacitors of ZEP520a containing PCBM with excellent retention characteristics, while negligible |Δ*V*_*F*_|’s were observed for those of ZEP520a without PCBM. Regarding the resist patterning characteristics, submicron or nanometer lateral-scale structures were fabricated simply by EB exposure on the ZEP520a containing PCBM and subsequent development. These results demonstrate the feasibility of not only submicron or nanometer lateral-scale memory cells but also electrical wires with submicron or nanometer widths, single-electron or quantum devices, or biosensors with nanostructures consisting of nanocomposite organic EB resist polymers containing fullerene molecules.

## Results

### Capacitance-voltage characteristics

**Figure 1:**
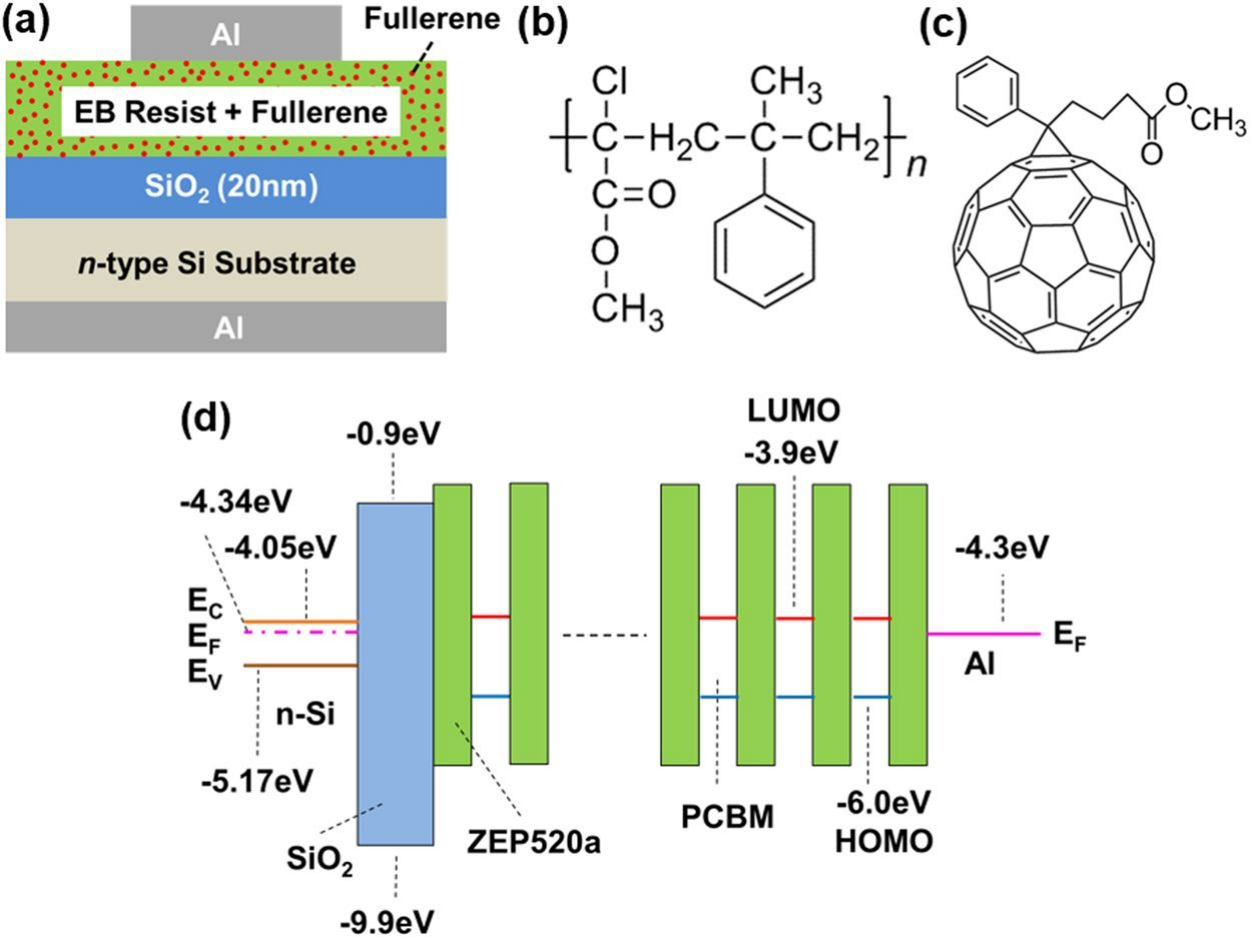
(**a**) Schematic diagram of fabricated capacitor with Al/electron-beam (EB) resist polymer containing fullerene/SiO_2_/*n*-type Si substrate. Chemical structure of (**b**) ZEP520a and (**c**) [6,6]-phenyl-C_61_ butyric acid methyl ester (PCBM). (**d**) Band diagram of nanocomposite resist polymer containing PCBM in the equilibrium state.

Figure 1a is a schematic diagram of a capacitor structure fabricated for the capacitance-voltage (*C*-*V*) measurements. A ZEP520a layer containing PCBM was formed on a 20-nm SiO_2_ layer on an *n*-type Si substrate (8–12 Ωcm). Figure 1b,c shows the molecular structures for ZEP520a and PCBM. The mole ratio of the fullerene relative to the monomer of ZEP520a was made to be 1:10. No EB exposures were performed for the samples in the *C*-*V* measurements, but the same development process as for the samples in the EB exposure experiments was carried out before depositing the Al gate and back electrodes. It should be noted that even if the nanometer-scale memory cell is fabricated using the ZEP520a containing PCBM proposed in this study, the remaining resist region after development, which is not exposed to electron beams, becomes a gate insulator that contains traps for carriers. The thickness of the resist was measured after development to be 150 nm by using spectroscopic ellipsometry. Capacitors of ZEP520a without PCBM were also fabricated for the purpose of comparison. The thickness of the resist after development was measured to be 120 nm. Figure 1d shows the band diagram of ZEP520a containing PCBM molecules with an Al gate electrode in the equilibrium state under the assumption that the PCBM molecules are uniformly dispersed. The indicated HOMO and LUMO levels of PCBM were determined from a survey of the literature^32^. The Fermi energy (*E*_*F*_) of the Si substrate was calculated using the doping level.

**Figure 2:**
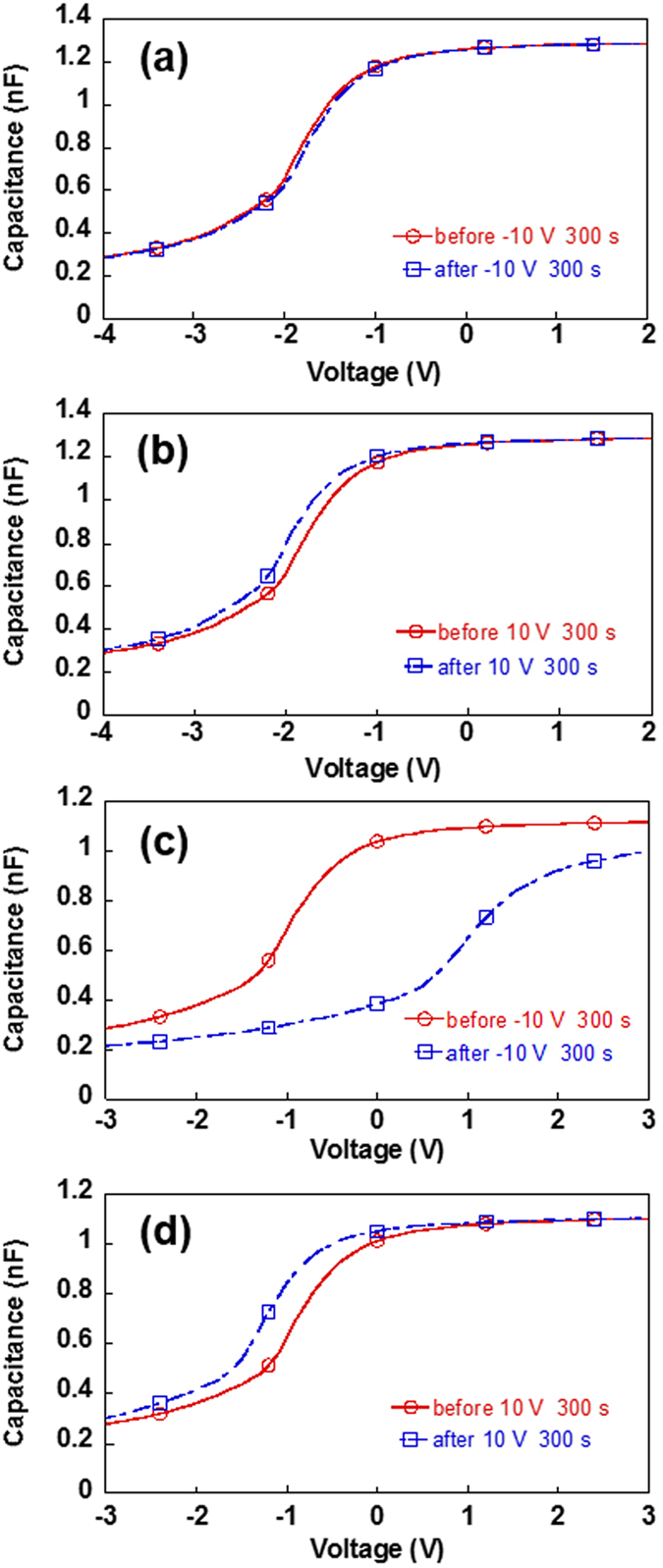
*C*-*V* programming characteristics at room temperature for the capacitor of ZEP520a without fullerene after writing at (**a**) −10.0 V and (**b**) 10.0 V for 300 s and for the capacitor of ZEP520a containing PCBM after writing at (**c**) −10.0 V and (**d**) 10.0 V for 300 s. Solid lines are for before applying the gate voltage, and dash-dotted lines are for after applying the gate voltage.

Figure 2 shows the *C*-*V* characteristics of the fabricated capacitors. The *C*-*V* curves were measured immediately after the gate voltage applications of ±10.0 V for 300 s. For the capacitors of ZEP520a without PCBM, a negligible Δ*V*_*F*_ smaller than 0.1 V was observed after −10.0 V was applied (Fig. 2a), and a very small Δ*V*_*F*_ of −0.2 V was observed after 10.0 V (Fig. 2b). The slightly negative Δ*V*_*F*_ after the positive voltage application may be due to the existence of a few bulk traps or interface traps between the ZEP520a layer and the Al gate electrode for holes. Trap levels are considered to exist below the *E*_*F*_ of the gate Al. For the capacitors of ZEP520a containing PCBM, on the other hand, a large Δ*V*_*F*_ of 2.4 V was observed after applying −10.0 V (Fig. 2c) while a small Δ*V*_*F*_ of −0.4 V was observed after applying +10.0 V (Fig. 2d).

The magnitude of effective injected charge |*Q*| is approximately given by ref. 33

1 $$|Q|={C}_{i}|{\rm{\Delta }}{V}_{F}|$$

Here, *C*_*i*_ is the insulator capacitance. Using this formula, the magnitude of effective negative charge (charge concentration) injected into the capacitor of ZEP520a without PCBM was less than 1.3 × 10^−10^ C (1.4 × 10^−4^ Ccm^−3^) after applying −10.0 V, while an effective positive charge of 2.5 × 10^−10^ C (2.7 × 10^−4^ Ccm^−3^) was injected after applying +10.0 V. An effective negative charge of −2.7 × 10^−9^ C (−2.5 × 10^−3^ Ccm^−3^) was injected into the capacitors of ZEP520a containing PCBM after applying −10.0 V, while an effective positive charge of 4.4 × 10^−10^ C (4.2 × 10^−4^ Ccm^−3^) was injected after applying +10.0 V. Here, the effective charge concentrations were calculated from the volumes of the resists in the capacitors.

The large |Δ*V*_*F*_| for the ZEP520a containing PCBM (Fig. 2c) in contrast to the negligible |Δ*V*_*F*_| for the ZEP520a without PCBM (Fig. 2a) after applying −10.0 V for 300 s indicates that electrons were injected into and stored in or around the PCBM molecules in the ZEP520a containing PCBM. Moreover, the much larger |Δ*V*_*F*_| after applying a negative programming voltage (−10.0 V for 300 s) than after a positive programming voltage (10.0 V for 300 s) for the ZEP520a containing PCBM (Fig. 2c,d) suggests that electrons are mainly injected into and stored in the LUMO levels of PCBM. The experimental programming characteristics reflect the relative energy difference between the HOMO or LUMO level of PCBM and the *E*_*F*_ of the gate Al. Because the LUMO level of PCBM is closer to the *E*_*F*_ of the gate Al than the HOMO level of PCBM is (Fig. 1d), electrons are preferentially injected into the LUMO levels of PCBM at negative programming voltages, while holes are difficult to inject into the HOMO levels even at positive programming voltages. Density functional theory (DFT) calculations^32^ indicate that the molecular orbitals in the LUMO region of PCBM are mainly derived from the orbitals of the C_60_ backbone. The small Δ*V*_*F*_ of −0.4 V that was observed after applying a positive programming voltage (10.0 V for 300 s) to the ZEP520a containing PCBM (Fig. 2d) suggests the existence of a few interface traps between the PCBM molecules and the matrix polymer of ZEP520a, since the |Δ*V*_*F*_| of 0.4 V is slightly larger than that of 0.2 V for the ZEP520a without PCBM (Fig. 2b). A few holes were probably injected into and stored in the interface trap levels.

*C*-*V* hysteresis traces were obtained for different minimum and maximum applied gate voltages (Figure S1). *C*-*V* curves for the ZEP520a containing PCBM shifted mainly in the positive voltage direction and |Δ*V*_*F*_| increased with increasing absolute value of the minimum and maximum gate voltages. Since a larger |Δ*V*_*F*_| indicates a larger amount of effective charge was injected, the result also indicates easy injection of electrons from the Al gate. Moreover, *C*-*V* hysteresis traces were obtained for different scanning rates of the gate voltage (Figure S2). *C*-*V* curves for the ZEP520a containing PCBM shifted mainly in the positive voltage direction and |Δ*V*_*F*_| increased with decreasing scanning rate, indicating a larger amount of effective charge was injected with a slower scanning rate because the duration of the applied voltage is longer for a slower scanning rate. These results indicate easy injection of electrons from the Al gate and support the interpretation that electrons are mainly injected into and stored in the LUMO levels of PCBM because of the relative energy difference (Fig. 1d) between the HOMO or LUMO level of PCBM and the *E*_*F*_ of the gate Al (see Supplementary Information, Section 1).

**Figure 3:**
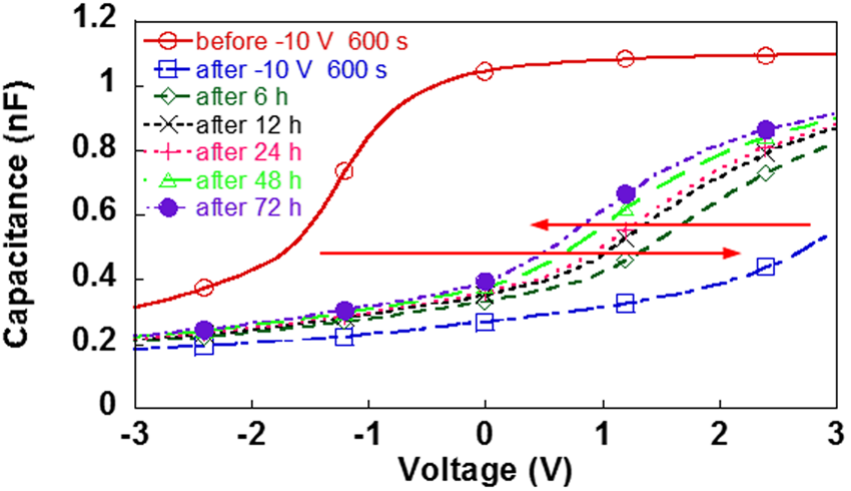
*C*-*V* retention characteristics for the capacitor of ZEP520a containing PCBM after writing at −10.0 V for 600 s. Arrows indicate the directions in which *C*-*V* curves shifted. Solid lines are for before applying the gate voltage, and dashed and dash-dotted lines are for after applying the gate voltage.

A large Δ*V*_*F*_ (effective injected charge and charge concentration) of more than 4 V (−4.4 × 10^−9^ C and −4.2 × 10^−3^ Ccm^−3^) and excellent retention characteristics were obtained after applying −10.0 V for 600 s to the ZEP520a containing PCBM (Fig. 3). About 50% of |Δ*V*_*F*_| remained three days later, compared with the value obtained immediately after programming.

### Exposure experiments

**Figure 4:**
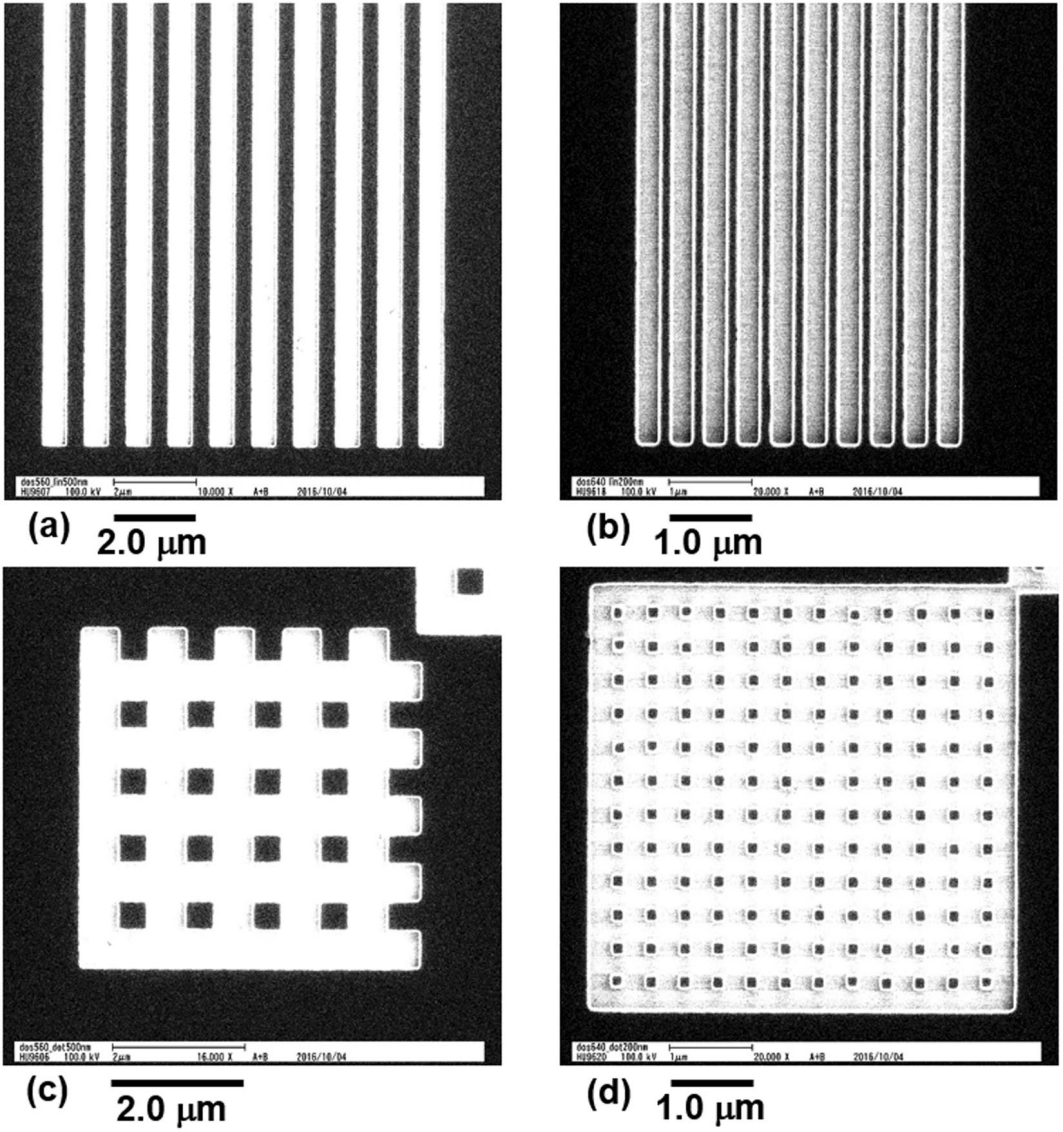
Plan-view SEM micrographs of line and space and square patterns for ZEP520a containing PCBM. The mask patterns of the lines and spaces have a line width of (**a**) 500 nm and (**b**) 200 nm, and the square mask patterns have a side length of (**c**) 500 nm and (**d**) 200 nm. Area doses are 560 μCcm^−2^ in (**a**) and (**c**) and 640 μCcm^−2^ in (**b**) and (**d**).

Next, exposure experiments on the resist of ZEP520a containing PCBM were carried out using an EB machine (ELS-G100 from ELIONIX) with an acceleration voltage of 100 kV. The same resist solution as used in the *C*-*V* measurements was spin-coated on a SiO_2_ layer and baked under the same conditions as in the *C*-*V* measurements (165 °C for 10 min). After the EB exposure, samples were developed in the ZEP developer ZED-N50 for 1 min and then IPA for 20 s with subsequent dipping into deionized water. Figure 4 shows plan-view scanning electron microscopy (SEM) images of line and space and square patterns. The mask patterns were lines and spaces with a line width of 500 nm and a pitch of 1000 nm (Fig. 4a) or with a line width of 200 nm and a pitch of 400 nm (Fig. 4b) and squares with a side length of 500 nm and a pitch of 1000 nm (Fig. 4c) or with a side length of 200 nm and a pitch of 400 nm (Fig. 4d). Area doses in Fig. 4a,c were 560 μCcm^−2^, while those in Fig. 4b,d were 640 μCcm^−2^. The white regions in the images are those in which the nanocomposite resist films had been removed, and the black regions are those in which the resist films remained after the development. The widths of the lines were about 400 nm for the 1000-nm-pitch mask patterns (Fig. 4a) and about 100 nm for the 400-nm-pitch mask patterns (Fig. 4b). The side lengths of the square were 370 nm for the 1000-nm-pitch square mask patterns (Fig. 4c) and about 100 nm for the 400-nm-pitch mask patterns (Fig. 4d).

**Figure 5:**
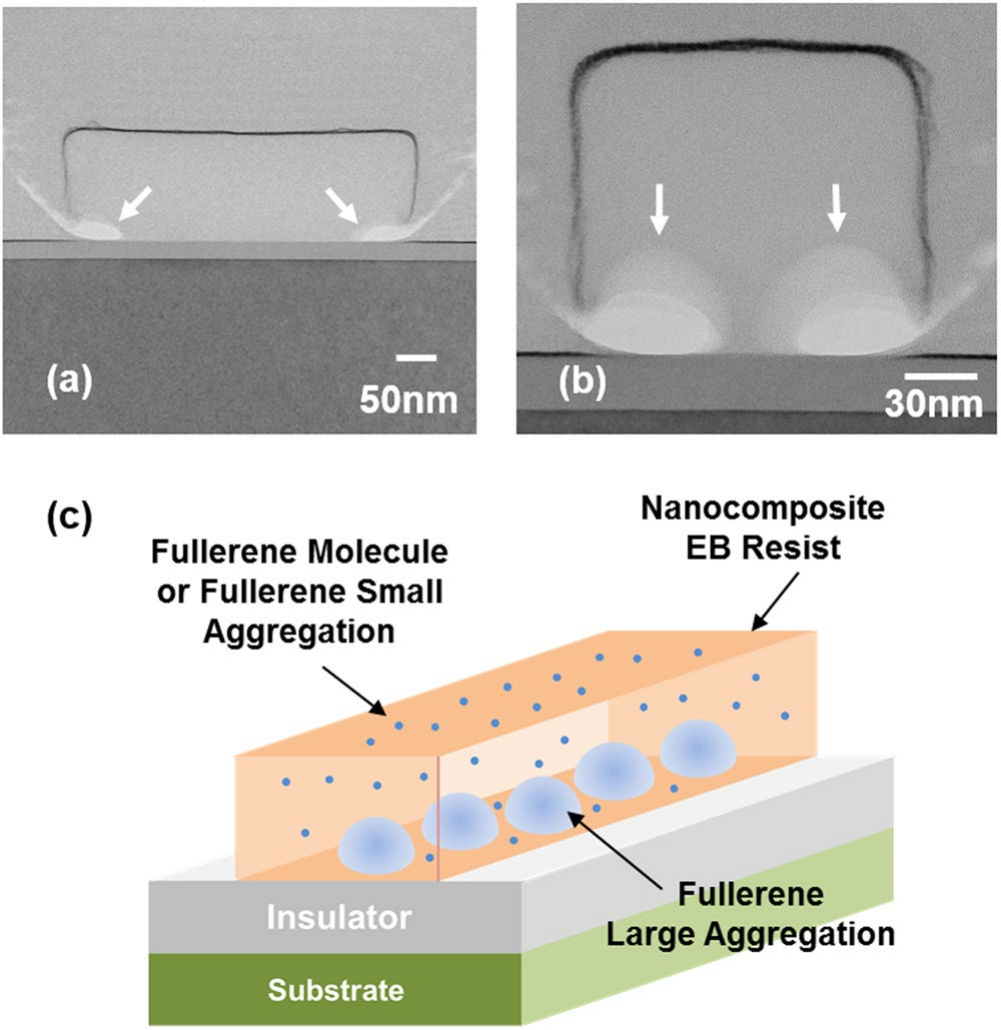
Cross sectional TEM micrograph of ZEP520a containing PCBM for mask patterns of lines and spaces with a line width of (**a**) 500 nm and (**b**) 200 nm. (**c**) Schematic diagram of the predicted distribution of PCBM molecules and aggregations in line pattern with a nanometer width of ZEP520a containing PCBM.

Figure 5a,b shows cross-sectional transmission electron microscopy (TEM) images of ZEP520a containing PCBM for the line and space patterns. Very clear patterns were formed from both mask patterns. For the 1000-nm-pitch patterns (Fig. 5a), the thickness and width of the formed line were 140 nm and 430 nm, respectively. No resist remained in the space regions on the SiO_2_ layer where the EB exposures occurred. For the 400-nm-pitch patterns (Fig. 5b), the thickness and width of the formed line were 130 nm and 140 nm, respectively. No resist remained in the space regions, either.

There are two white regions, indicated by arrows, near the bottom edges of each resist pattern in Fig. 5a,b. These regions are empty, and no resist or PCBM exists. In contrast, there are no white regions in the upper region of the resist patterns. These results suggest that the concentration of PCBM was higher in the bottom region of the resist and also that the PCBM molecules aggregated there, judging from the white semispherical contrasts indicated by the arrows. Because the concentration of ZEP520a was low in these regions, rapid etching occurred there during the development with ZED-N50 and IPA. The base diameters of the semispherical PCBM aggregation are about 60 nm, and the base center-to-center distance of the adjacent aggregations is about 80 nm in Fig. 5b. Accordingly, several semispherical PCBM aggregations are speculated to exist in the inner bottom region of the resist line pattern with the larger width (Fig. 5a). Because these PCBM aggregations are considered to be amorphous without crystal lattice structures, it is impossible to distinguish them from the surrounding resist materials. Note that the concentration of PCBM in the upper region of the resist pattern was lower than that in the bottom region, judging from the fact that much less resist had been removed from the upper surface of the resist than from the edge bottom region after development (the resist thickness of ZEP520a containing PCBM measured by using spectroscopic ellipsometry decreased to 150 nm from 160 nm before development of the *C*-*V* samples). The thickness reduction of the resist is related to the existence of PCBM in ZEP520a because the thickness of ZEP520a without PCBM did not change (120 nm for the *C*-*V* samples) after development. Dispersed PCBMs in the upper region are considered to be in a molecular state or in much smaller aggregations with a lower concentration than those of the bottom region (Fig. 5c). Judging from the base diameter and center-to-center distance of these large bottom PCBM aggregations, the minimum side length of a memory cell or the minimum width of a conducting line would be about 200 nm. Optimization of the kind and concentration of incorporated fullerene as well as of the baking conditions of the resist will make it possible to reduce the size of the fullerene aggregation, leading to a further reduction in lateral device size.

## Discussion

The above predicted distribution of PCBM molecules or aggregations in ZEP520a containing PCBM would be advantageous for lengthening the memory retention time because the charging energy of the larger PCBM aggregates is smaller than that of PCBM molecules or the smaller aggregates. Actually, an extra electron of charge -*e* injected into a PCBM aggregate increases the electrostatic potential energy by the charging energy^34^,

2 $${E}_{C}={e}^{2}/{C}_{S}$$

Here, *C*_*S*_ and *e* are the self-capacitance of the aggregate and the elementary charge, respectively. Consequently, the smaller the PCBM aggregate is, the larger (smaller) the *E*_*C*_ (*C*_*S*_) becomes. Therefore, once electrons are injected into the large PCBM aggregates near the bottom of the resist, they find it difficult to return to the gate electrode. This may be the reason for the extremely long retention time of the ZEP520a containing PCBM shown in Fig. 3 (see Supplementary Information, Section 2).

On the other hand, when the resist is used for electrical wiring with a submicron or nanometer width, a large current will flow through the bottom region because of the high concentration of PCBM and the smaller charging energies of the large semispherical PCBM aggregations. Moreover, there is the possibility of observing at low temperatures single electron effects such as Coulomb oscillations because the large PCBM aggregations become Coulomb islands. The necessary condition to observe a single electron effect is ref. 34

3 $${e}^{2}/{C}_{S} > {k}_{B}T$$

Here, *k*_*B*_ and *T* are the Boltzmann constant and observing temperature, respectively. Since the diameters of the large PCBM aggregations are about 60 nm (Fig. 5b), single electron effects will be observed below 200 K.

Furthermore, there is a possibility that the fullerene aggregations can serve as building blocks to form a solid-state-based qubit for quantum information processing^12^. The technique proposed in this study would make it possible to arrange a large number of qubits at precisely determined positions. In these cases, it is possible to reduce the decoherence of the electron spin via hyperfine interactions with nuclear spins in the fullerene aggregation because group IV materials such as carbon^35, 36^, Si^37^, and Si-Ge^38^, abundantly comprise isotopes free of nuclear spins. Moreover, if an electrical wire having nanometer width is used for the channel region of a field effect transistor biosensor or a single-electron transistor is used for a biosensor, the sensitivity of detection increases when the target species are diluted in concentration because of the reduction of the effect of carrier percolation in the channel^8, 9, 11^ or the effect of Coulomb oscillation^10, 11^.

## Conclusion

In conclusion, ZEP520a containing PCBM was proved to be an excellent candidate for an electrically conductive and/or memory component material for submicron or nanometer lateral-scale electronic devices, especially for submicron lateral-scale memory cells. The carrier transfer and retention mechanisms were investigated for the nanocomposite EB resist polymer. Line patterns (square patterns) of ZEP520a containing PCBM can be made with widths (side lengths) of less than 200 nm by using an extremely simple process with only EB exposures and developments. The distribution of PCBM molecules or their aggregations was clarified in the nanocomposite resist. These results open the door to a simple way of fabricating organic ultrahigh integrated flexible memories, electrical wires, and single-electron or quantum devices including quantum information devices. The technique proposed in this study is also applicable to high sensitive biosensors having submicron or nanometer width channels and/or Coulomb islands in organic integrated single-chip systems suitable for multiplexed and simultaneous diagnoses.

## Methods

### Device Fabrication

A conventional positive-type electron beam resist, ZEP520a, was chosen as the matrix polymer of a nanocomposite organic EB resist polymer. ZEP520a and PCBM were purchased from Nippon Zeon Co. and Sigma-Aldrich Co., respectively. In the sample fabrication of ZEP520a containing PCBM, PCBMs were dissolved in a ZEP520a resist solution with an anisole solvent, and the mixture was ultrasonicated for 6 h to obtain a uniform solution before spin coating. The mole ratio of the fullerene relative to the monomer of ZEP520a was made to be 1:10. The resist solution was spin coated on the SiO_2_ layer with a thickness of 20 nm grown on an *n*-type (P-doped) Si substrate (8–12 Ωcm) by dry oxidation and then baked at 165 °C for 10 min. No EB exposures were performed for the samples in the *C*-*V* measurements but the same development process as for the samples in EB exposure experiments was carried out before depositing the Al gate and back electrodes. Al gate electrodes were deposited using a metal stencil mask for capacitors. The area of an Al gate electrode was 0.071 cm^2^.The capacitors of ZEP520a without PCBM were also fabricated for the purpose of comparison. The ZEP520a resist (without PCBM) with an anisole solvent was ultrasonicated for 6 h. The resist solution was spin coated on the SiO_2_ layer and baked at 165 °C for 10 min. No EB exposures were performed but the same development process was carried out before depositing the Al electrodes. For exposure experiments, the same resist solution of ZEP520a containing PCBM as used in the *C*-*V* measurements was spin-coated on a SiO_2_ layer and baked under the same conditions as in the *C*-*V* measurements (165 °C for 10 min). EB exposures on the resist of ZEP520a containing PCBM were carried out using an EB machine (ELS-G100 from ELIONIX) with an acceleration voltage of 100 kV. After the EB exposure, samples were developed in the ZEP developer ZED-N50 for 1 min and then IPA for 20 s with subsequent dipping into deionized water.

### Characterization

*C*-*V* curves were monitored at 50 kHz in a dark environment at room temperature. In programming and retention characteristics, *C*-*V* curves were obtained with a voltage scan from a maximum gate voltage to a minimum gate voltage. There was no time delay between setting the gate voltage and making the capacitance measurement. Δ*V*_*F*_’s were evaluated from the *C*-*V* curves at the flatband capacitances. In the fabricated structures, the Δ*V*_*F*_’s are opposite to those in the conventional flash memories, where charge injection occurs from Si; the reason is charge injection from the metal gate side. Plan-view SEM images were captured using the ELS-G100 with an acceleration voltage of 100 kV. Cross-sectional TEM images were captured using an H-9500 from Hitachi High-Technologies with an acceleration voltage of 200 kV.

## Electronic supplementary material

Supplementary Information
