# Supplementary material for: Fullerene-Containing Electrically Conducting Electron Beam Resist for Ultrahigh Integration of Nanometer Lateral-Scale Organic Electronic Devices
Source: Sci Rep. 2017 Jun 27;7:4306. doi: 10.1038/s41598-017-04451-9 (PMC5487332; doi:10.1038/s41598-017-04451-9)
Supplement: Supplementary file 1 — Supplementary Information [file 41598_2017_4451_MOESM1_ESM.pdf]

Supplementary Information

Fullerene-Containing Electrically Conducting Electron Beam Resist for Ultrahigh Integration of Nanometer Lateral-Scale Organic Electronic Devices

Anri Nakajima,* Tetsuo Tabei & Tatsuya Yasukawa

Research Institute for Nanodevice and Bio Systems, Hiroshima University, 1-4-2 Kagamiyama, Higashihiroshima, Hiroshima 739-8527, Japan

*E-mail: anakajima@hiroshima-u.ac.jp

The supplementary information contains the following sections:

1. Capacitance-voltage hysteresis characteristics

2. Capacitance-voltage retention characteristics

1. Capacitance-voltage hysteresis characteristics

Capacitance-voltage (*C*-*V*) hysteresis characteristics were measured in order to examine the memory mechanism of the nanocomposite resist proposed in this study. A ZEP520a layer containing PCBM was formed on a 20-nm SiO_2 layer on an *n*-type Si substrate (8–12 Ωcm). The mole ratio of the fullerene relative to the monomer of ZEP520a was made to be 1:10. Capacitors of ZEP520a without PCBM were also fabricated for the purpose of comparison. No EB exposures were performed for the samples, but the development process was carried out before depositing the Al gate and back electrodes. The ZEP520a containing PCBM and ZEP520a without PCBM of the capacitors used in the *C*-*V* hysteresis measurements were respectively 130 nm and 120 nm thick, as measured with spectroscopic ellipsometry after development.

Figure S1 shows *C*-*V* hysteresis traces for different minimum and maximum applied gate voltages. The *C*-*V* curves were obtained with an initial voltage scan from the maximum gate voltage to the minimum gate voltage and a subsequent scan in the opposite direction. There was no time delay between setting the voltage and making the capacitance measurement. For the capacitor made of ZEP520a without PCBM (Fig. S1a,b), the *C*-*V* curves shifted slightly in the positive gate voltage direction after the subsequent voltage scan in the positive voltage direction with a small flat band voltage shift (ΔV_F) of about 0.1 V even after large-magnitude ($\pm$10.0 V) minimum and maximum voltages were applied (Fig. S1b). For the capacitor made of ZEP520a containing PCBM (Fig. S1c), on the other hand, a substantial $|\Delta V_F|$ (0.2 V) was observed even after much smaller magnitude ($\pm$3.0 V) minimum and maximum voltages were applied. The *C*-*V* curves shifted mainly in the positive voltage direction after the subsequent voltage scan in the positive voltage direction, and $|\Delta V_F|$ increased with increasing magnitude of minimum and

maximum applied gate voltage. A larger $|\Delta V_F|$ indicates a larger amount of effective charge was injected. Since the shifts of the *C*-*V* curves in the positive directions occurred after application of negative gate voltages, the results indicate easy injection of electrons from the Al gate and support the interpretation that electrons are mainly injected into and stored in the LUMO levels of PCBM because of the relative energy difference between the HOMO or LUMO level of PCBM and the Fermi energy E_F of the gate Al (Fig. 1d).

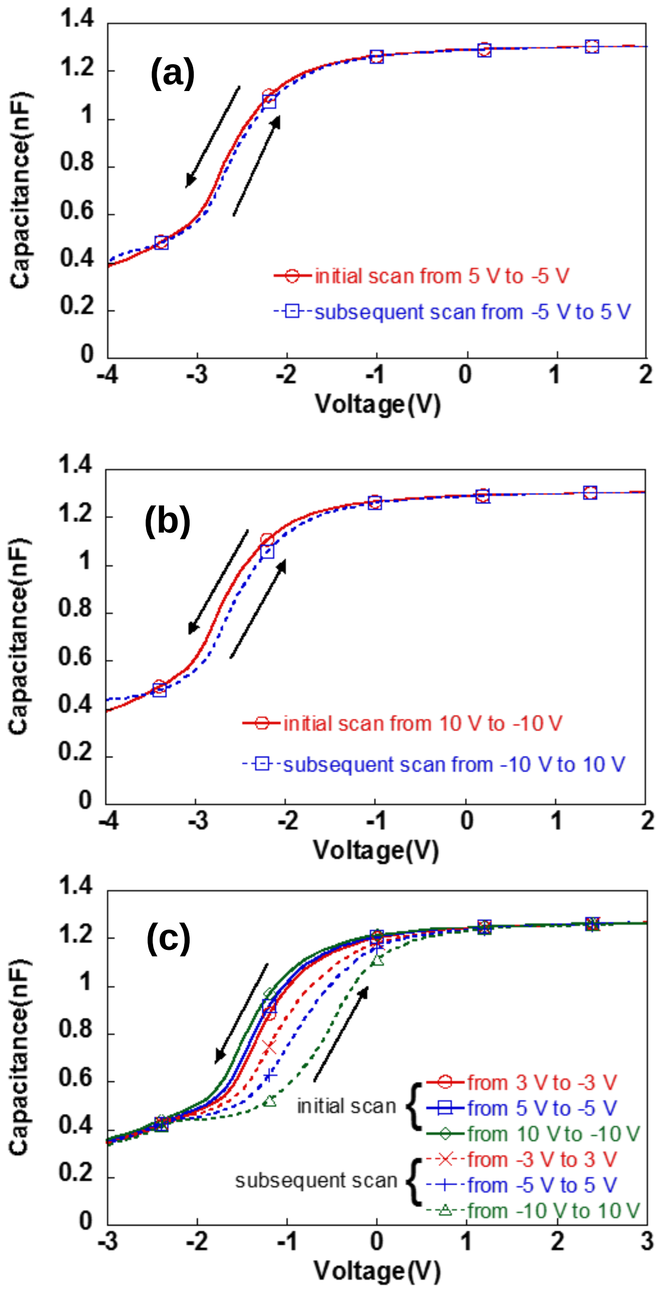

Figure S1. *C*-*V* hysteresis traces for different minimum and maximum applied gate voltages (a,b) for a capacitor of ZEP520a without fullerene and (c) for a capacitor of ZEP520a containing PCBM. *C*-*V* curves were obtained with an initial voltage scan (solid lines) from the maximum gate voltage to the minimum gate voltage and a subsequent scan in the opposite direction (dashed lines). There was no time delay between setting the voltage and making the capacitance measurement.

Figure S2 shows *C*-*V* hysteresis traces made with different voltage scanning rates. The *C*-*V* curves were obtained with an initial voltage scan from the maximum gate voltage to the minimum gate voltage and a subsequent scan in the opposite direction. For the capacitor made of ZEP520a without PCBM (Fig. S2a,b), ΔV_F showed almost no scanning rate dependence. ΔV_F was negligible (less than 0.05 V) in both cases of a fast voltage scan with no time delay between setting the voltage and making the measurement (Fig. S2a) and a slow voltage scan with a time delay of 1 s (Fig. S2b). For the capacitor made of ZEP520a containing PCBM (Fig. S2c), on the other hand, the *C*-*V* curves shifted mainly in the positive voltage direction after the subsequent voltage scan in the positive voltage direction and $|\Delta V_F|$ increased with decreasing scanning rate. A larger $|\Delta V_F|$ indicates a larger amount of effective charge was injected. Since the shifts of the *C*-*V* curves in the positive directions occurred after application of negative gate voltages and the time of the applied negative voltage increases with increasing time delay (decreasing scanning rate), the results also indicate easy injection of electrons from the Al gate and support the interpretation that electrons are mainly injected into and stored in the LUMO levels of PCBM.

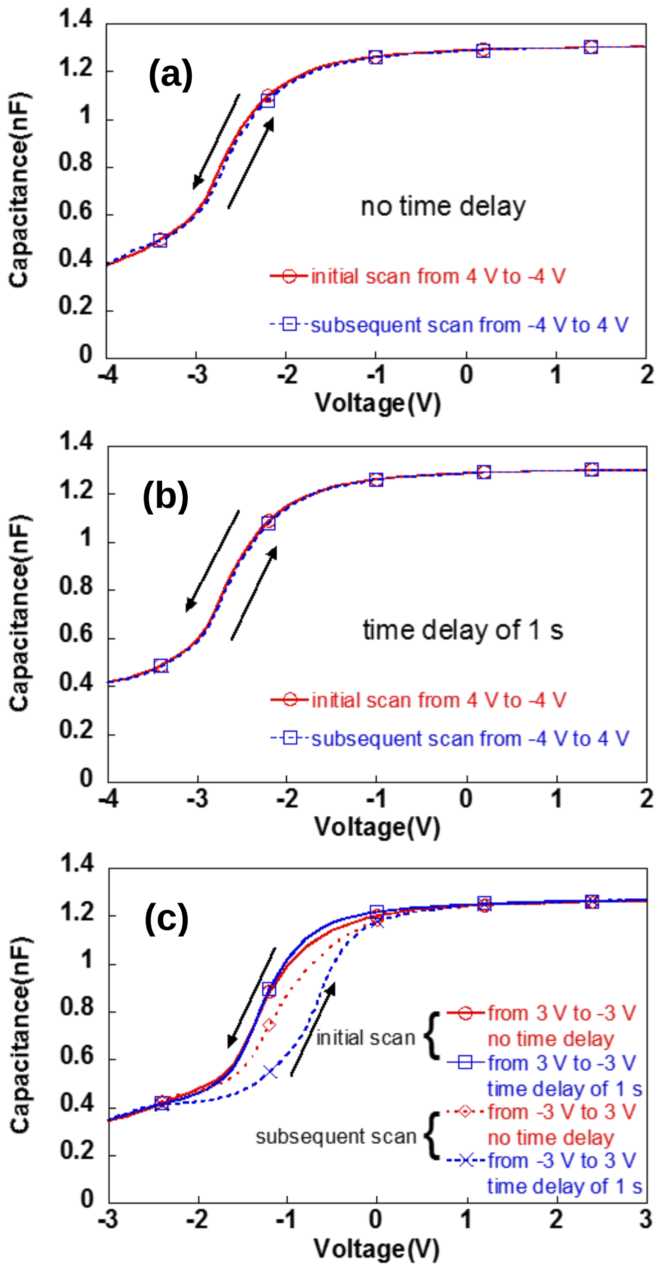

Figure S2. *C-V* hysteresis traces made with different voltage scanning rates (a,b) for capacitors composed of ZEP520a without fullerene and (c) for capacitors composed of ZEP520a containing PCBM. *C-V* curves were obtained with an initial voltage scan (solid lines) from the maximum gate voltage to the minimum gate voltage and a subsequent scan in the opposite direction (dashed lines). Fast gate voltage scans were carried out with no time delay between applying the gate voltage and making the measurement and slow gate voltage scans with a time delay of 1 s.

2. Capacitance-voltage retention characteristics

Figure 3 shows that the retention characteristics consist of components with short retention times and those with long retention times. This can be understood, for example, by the fact that $|\Delta V_F|$ after the first 12 hours is much larger than it is after the next 12 hours. The components with short retention times are considered to arise from PCBM in a molecular state or in small aggregations around the top of the nanocomposite resist, while the components with long retention times arise from the large PCBM aggregates near the bottom. Thus, the large PCBM aggregates near the bottom are considered to control the overall retention time.
